# Array of Chemosensitive Resistors with Composites of Gas Chromatography (GC) Materials and Carbon Black for Detection and Recognition of VOCs: A Basic Study

**DOI:** 10.3390/s17071606

**Published:** 2017-07-11

**Authors:** Bartosz Wyszynski, Rui Yatabe, Atsuo Nakao, Masaya Nakatani, Akio Oki, Hiroaki Oka, Kiyoshi Toko

**Affiliations:** 1 Research and Development Center for Taste and Odor Sensing, Nishi-ku, Motooka 744, Fukuoka 819-0395, Japan; toko@ed.kyushu-u.ac.jp; 2 Panasonic Automotive & Industrial Systems Company, Sensing Solutions Development Center, Kadomoa-shi, Oaza Kadoma 1006, Osaka 571-8501, Japan; nakao.atsuo@jp.panasonic.com (A.N.); nakatani.masaya@jp.panasonic.com (M.N.); oki.akio@jp.panasonic.com (A.O.); oka.hiroaki@jp.panasonic.com (H.O.)

**Keywords:** odor sensor, chemical sensor, artificial olfaction, chemoresistance, sensor array, GC material, carbon black

## Abstract

Mimicking the biological olfaction, large odor-sensor arrays can be used to acquire a broad range of chemical information, with a potentially high degree of redundancy, to allow for enhanced control over the sensitivity and selectivity of artificial olfaction systems. The arrays should consist of the largest possible number of individual sensing elements while being miniaturized. Chemosensitive resistors are one of the sensing platforms that have a potential to satisfy these two conditions. In this work we test viability of fabricating a 16-element chemosensitive resistor array for detection and recognition of volatile organic compounds (VOCs). The sensors were fabricated using blends of carbon black and gas chromatography (GC) stationary-phase materials preselected based on their sorption properties. Blends of the selected GC materials with carbon black particles were subsequently coated over chemosensitive resistor devices and the resulting sensors/arrays evaluated in exposure experiments against vapors of pyrrole, benzenal, nonanal, and 2-phenethylamine at 150, 300, 450, and 900 ppb. Responses of the fabricated 16-element array were stable and differed for each individual odorant sample, proving the blends of GC materials with carbon black particles can be effectively used for fabrication of large odor-sensing arrays based on chemosensitive resistors. The obtained results suggest that the proposed sensing devices could be effective in discriminating odor/vapor samples at the sub-ppm level.

## 1. Introduction

Airborne chemicals carry an enormous amount of information about both narrowly and broadly defined environments. Biological sense of smell is naturally employed to such diverse tasks as early warning of possible dangers, presence of certain health conditions, food safety, or simple indulgence of aromas [1]. Biological olfaction has been widely applied in various fields for either expert or non-expert evaluations, using techniques of sensory analysis [2], often combined with instrumental analytical methods such as spectroscopy or chromatography [3]. However, both sensory and instrumental analyses can be quite impractical due to their costs and time and space limitations as well as their lack of mobility.

Artificial olfaction is a relatively novel field, taking advantage of our understanding of biological olfaction. Artificial olfaction instruments—electronic noses—emerge as an attractive alternative to sensory and instrumental analyses as they can overcome most or all of the downsides listed above [4,5]. Similar to its biological counterpart, an electronic nose makes sense of information acquired from an array of odor-receptive devices—gas/vapor sensors. Similar to the olfactory receptors, the sensors should be non-specific, i.e., respond to groups of odorants rather than a particular one, and such broad tuning should overlap across the array. In the following step, such non-specific responses are subject of further processing using various pattern recognition techniques—from quite simple principal component or linear discriminant analyses to neural networks [6,7,8,9,10]. This combinatorial approach allows for tuning of overall system performance in terms of specificity by choosing appropriate odor-interactive characteristics of the individual sensors making up the arrays.

Although quite well established, the electronic nose systems are subject of intensive studies with plethora of applications ranging from food and beverage industries to environmental monitoring to medical diagnostics [11,12,13,14,15,16,17].

Chemical sensors used in modern electronic nose systems can be classified in the following manner: metal oxide semiconductor (MOS), metal oxide semiconductor field-effect transistor (MOSFET), calorimetric, optical, quartz crystal microbalance (QCM), surface acoustic wave (SAW), conducting polymer, and carbon material composites (carbon nanotubes or carbon black particles) [14,18,19,20]. Summarized characteristics of the types of sensors, including their advantages and disadvantages, are presented in Table 1.

Two types of sensors described in Table 1—conducting polymers and carbon particle based sensors—often classified as volumetric sensors, are very well suited for use in the artificial olfaction due to their relatively simple structure and ease of fabrication and customization [21]. They also possess a great potential advantage of miniaturization which seems perfect for creation of sensor arrays—small in size but large in the number of sensing elements [22,23,24]. In brief, the volumetric devices consist of two electrodes and an analyte-interactive film capable of changing its volume upon sorption of analytes. Quite obviously, the film has to be electrically conductive to a certain degree. One way to achieve that is by application of intrinsically conductive materials such as conductive polymers [25,26]. Another way, exploited by our group, is to use composites/blends of conductive particles (e.g., carbon black or zinc oxide) with sorptive, insulating materials [27,28]. Such composite film is coated between the electrodes and electric current is drawn across them. In absence of an analyte the particles in the composite film form a conductive network which can be characterized by its conductivity/resistivity. Upon sorption of analyte the film undergoes a volumetric change (“swelling effect”) that increases distance between the conductive particles thus increasing resistance of the overall conductive composite [27].

As shown in Table 1, sensing films composed of carbon black are not the only kind of carbon based materials used in chemical sensors. A great wealth of studies have been devoted to other forms of carbon materials, for instance carbon nanotubes. Details concerning such materials and their applications are covered in a number of excellent reviews (e.g., [27,28,29]).

Sensing properties of a carbon black-containing composite can be tuned by choice of a particular sorptive material. Among many materials that could potentially be used as a sorptive part in the composite films, the most widespread seem to be polymers. Polymeric materials are rheologically stable which significantly simplifies their mixing with carbon black powders and subsequent application and evaluation. However, there are plethora of other materials with great gas-sorption properties that have not or seldom been used for volumetric sensors. A large group of such interesting materials are stationary phase materials used in gas chromatography (GC materials). From the standpoint of odor-sensing, the most important characteristic of a GC material seems to be the extent to which the material would interact with odorant molecules. Such characterization can be possible based on classic metrics used in gas chromatography—retention and McReynolds indices [30]. To be sure, both retention and McReynolds indices’ systems are subject of discussion (e.g., [31]) but they provide an important preliminary insight into potential material–analyte interaction. This insight can very well be used for tentative selection of GC materials to be used in odor-sensing composites.

In this paper, we present our basic study on selection and application of GC stationary phase materials as the sorptive (non-conductive) part in odor-sensing composites used in chemosensitive resistor-based odor sensors. The materials are tentatively selected based on known gas chromatography indices. Then their actual odor-sensing properties are evaluated experimentally using quartz crystal microbalance (QCM) sensors. After that, the selected materials are mixed with carbon black, the resulting composites coated onto chemoresistive devices, and their odor-sensing properties evaluated against vapors of formaldehyde, acetaldehyde, nonanal, benzenal, 2-phenethylamine, and pyrrole.

To the best of our knowledge, application of GC stationary phase materials blended with carbon black has not been reported to date. In our view, the main advantage of using such blends stems manly from three facts: (i) GC stationary phase materials are readily available in great variety; (ii) preparation of composites/blends with carbon black is relatively simple; (iii) properties of the composites/blends can potentially be tuned toward particular odor sensing tasks.

## 2. Materials and Methods

21 stationary phase GC materials were preselected for use in this study based on their known chromatographic characteristics, indicating potential interaction of a material with classes of analytes. In our approach, the GC materials were chosen to cover the broadest possible range of the potential interaction with our analytes of choice. The materials were used as obtained and are listed along with their manufacturers in Table 2.

The odor-sensing properties of the preselected GC materials were evaluated using QCM devices. The QCMs used here were 20 MHz devices with gold, mirror-polished electrodes, obtained from Seiko, Co., harbour district, Japan. The GC materials were coated onto the QCMs from chloroform solutions (1 g/L) using a spray-coating technique. The amount of the coated materials was monitored during the coating process using universal frequency counter 53131A (Agilent, Santa Clara, CA, USA).

Conductive carbon black particles (graphite carbon black) were obtained from Sigma-Aldrich. In the preliminary viability tests, using a simple 1-channel chemoresistive device, the conductive composite materials were prepared by adding the carbon black particles (8 g/L) to chloroform solutions of the GC materials (8 g/L). In the further tests involving a 16-channel chemoresistive chip, the carbon black particles were mixed with the GC materials dissolved in DMSO (dimethylsulfoxide) or DMSO/MeCN.

Two types of the chemoresistive devices used in this work were fabricated by Panasonic. The first type was a simple 1-channel device composed of insulated circular electrodes with open contact points. The distance between the contact points was 50 µm. The second type was a 16-channel microdevice fabricated on a p-type Si monocrystalline substrate (8 × 8 mm^2^). Two platinum electrodes were formed as concentric circles using a photo lithography process. The distance between the electrodes was 50 µm to 280 µm.

The composite materials used in the viability study were coated onto the chemoresistive devices by manual spotting of the chloroform solutions/suspensions using micropipette. Prior to the coating the composite chloroform solutions/suspensions were ultrasonicated for at least 2 min to ensure proper mixing of carbon black with the GC materials. The composites used in the proper study (16-channel chips) were coated as a circle mark using a customized automatic spotting machine with a microsyringe. The volume of ejected solvents was 25 nL. The ejections were carried out 4 times for each mark. The diameter of spotted mark was 950 µm while the thickness of the deposited film was typically 700–800 nm.

**Figure 1:**
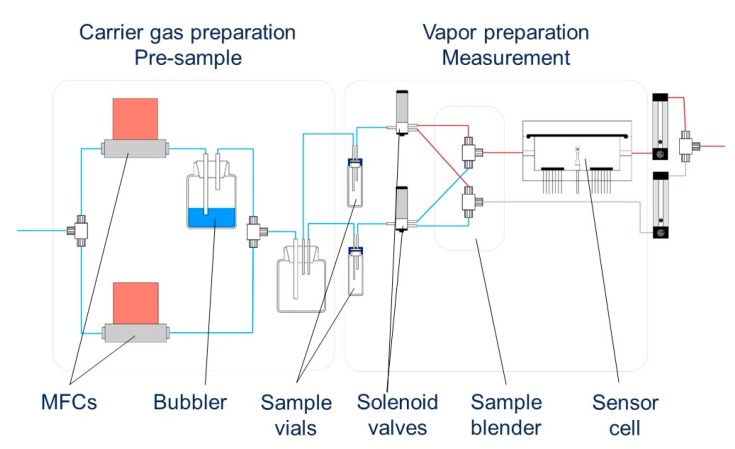
Schematic representation of the measurement system used in the study.

The fabricated odor-sensors (both QCM and chemosensitive resistors) were evaluated at room temperature in a measurement system shown in Figure 1. Liquid samples, dissolved with non-volatile and odor-free dioctanoyl decanoyl glycerol (ODO, 0.1%, *v*/*v*) were placed in vials and their dynamic headspace carried into a measurement chamber at 500 mL/min. Dry nitrogen was used as a carrier gas (no water in the bubbler). Streams of the sample and the carrier gas were alternated using solenoid valves.

The odor samples used in the QCM study were formaldehyde, acetaldehyde, nonanal, benzaldehyde, 2-phenethylamine, and pyrrole. Evaluation of the chemosensitive resistor devices was performed using nonanal, benzaldehyde, 2-phenethylamine, and pyrrole. The odorants were chosen mainly on the basis of their structure, i.e., we have chosen homologues of aliphatic aldehydes, 1 aromatic aldehyde, 1 aromatic amine, and 1 heterocyclic aromatic compound. The samples were purchased from Tokyo Kasei Co. and Sigma-Aldrich and were used as obtained.

The QCM and 1-channel chemosensitive resistor sensors were evaluated in the experiments starting with 60 s exposure to carrier gas, followed by 60 s exposure to analyte vapor, followed subsequently by 120 s exposure to carrier gas (recovery phase). The above exposure pattern was repeated thrice for each sensor and the experiments were performed at 4 levels of analyte concentration.

The 16-channel chemosensitive resistor sensors were evaluated in slightly modified experiments starting with 60 s exposure to carrier gas, followed by alternating 60 s exposures to analyte vapor and carrier gas (recovery phase). Each sensor was exposed thrice to analytes presented at 4 concentration levels (i.e., total of 12 exposures = 4 concentration levels × 3 exposures at each level).

Responses of the fabricated QCM and 1-channel chemosensitive resistor sensors were firstly prepared to account for possible baseline drift and then calculated as a difference between the sensor’s response at the sample’s onsets (60, 240, and 420 s, respectively) and the sensor’s response at the sample’s offsets (120, 300, and 480 s, respectively).

Similarly, responses of the 16-channel chemosensitive resistor sensors were calculated as difference between response at sample’s onsets (60, 180, 300 s, etc.) and offsets (120, 240, 360 s, etc.).

Capability of the fabricated sensors to discriminate among the odorant samples was evaluated using Principal Component Analysis (PCA) technique. PCA is a quite standard and simple pattern recognition technique that allows for reduction of data dimensionality.

In the PCA method, the information extracted from the multivariate data set is represented using a set of artificial variables called principal components. The principal components (or factors) are calculated by seeking maximum variance within the original data set with the assumption that each consecutive principal component axis is orthogonal to the previous one (e.g., Factor#1 orthogonal to Factor#2, Factor#2 orthogonal to Factor#3, etc.). The obtained sensor responses were first preprocessed by means of variance scaling to account for differences in sensor response magnitude. The preprocessed data matrix was then fed to the Principal Component Analysis (PCA) routine developed in-house.

## 3. Results and Discussion

### 3.1. Preselection of GC Materials Using QCM Sensors

Characteristics of the QCM sensors fabricated here are listed in Table 2, along with manufacturers of the coated GC materials.

The QCM sensors were fabricated using a simple spray-coating technique and their frequency measured right before and 24 h after coating (to allow for complete evaporation of chloroform). Many of the GC materials used in this part of the study are liquid at room temperature which is reflected in the varying amounts of coated material expressed as frequency shift due to coating. As the materials were coated directly onto the QCM device surface (mirror polished quartz crystals with Au electrodes), only the materials forming coherent, stable films could be deposited in larger amounts and conversely, the ones forming less stable films (liquid phase materials) couldn’t be coated in large amounts without compromising oscillation of the quartz resonators.

**Figure 2:**
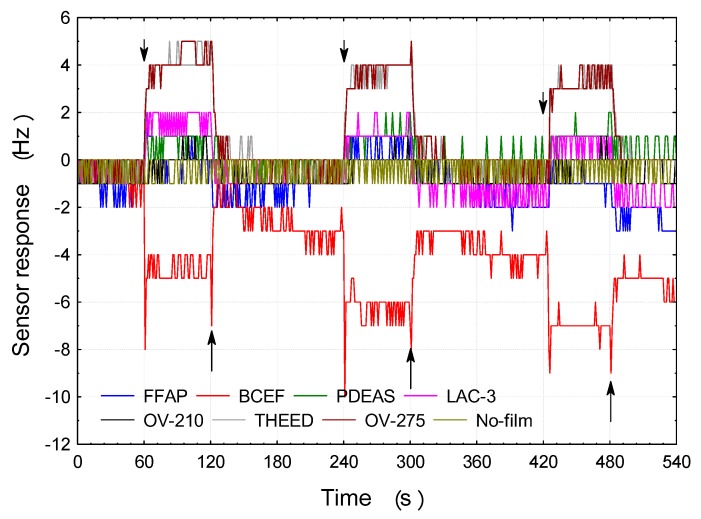
Transient responses of 7 QCM sensors to benzaldehyde at 250 ppb and 0% RH.

All fabricated sensors were subsequently evaluated in exposure experiments against vapors of 6 odorant samples. Each sensor was exposed to each vapor at ca. 250 ppb. As an example, transient responses of 7 sensors coated with GC materials to benzenal are shown in Figure 2. A non-coated QCM was included in the set for monitoring purposes. The down- and up-arrows denote onset and offset of the sample, respectively.

As can be seen, all the coated sensors responded to the presence of an odorant sample in a stable manner. Their responses were relatively fast and reversible, i.e., they were clearly returning to a baseline after the sample offset. The N,N-Bis(2-cyanoethyl)formamide (BCEF) sensor exhibited a slight drift of its baseline as well as “negative” response to the odorant sample—both phenomena attributable to the viscoelastic properties of the BCEF film.

The calculated responses were used to form a response matrix used for subsequent analysis. The data set of 6 samples triplicates (6 × 3 = 18) by 21 GC materials was first preprocessed to account for differences in sensor responses and then fed to the PCA routine developed in house.

**Figure 3:**
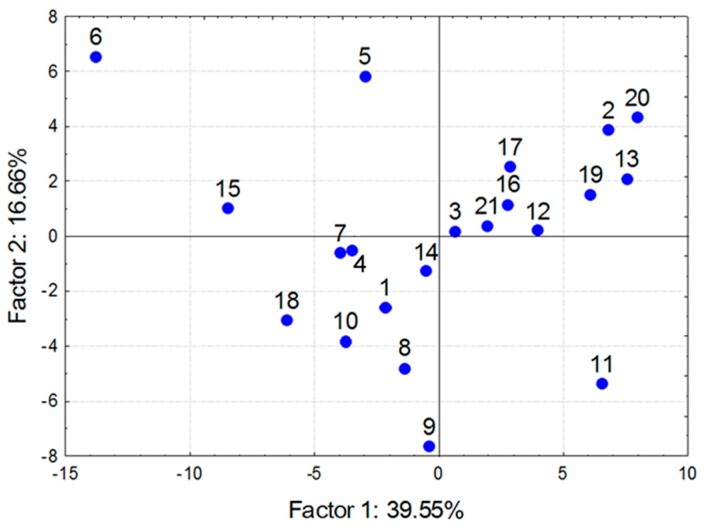
Principal component analysis (PCA) scattering of all 21 GC materials based on responses to triplicates of 6 odorant samples (21 sensors × 18 samples).

Firstly, the transposed data matrix was used to assess the odor-sensing properties of the 21 GC materials upon response to the 6 odorant samples. Obtained PCA scattering of Factor 1 vs. Factor 2 scores is shown in Figure 3.

The labels in the diagram refer to Table 1 and each one denotes an individual GC material. As can be seen the data points were scattered rather uniformly, without significant clustering. Visual inspection of the diagram in Figure 3 suggests that the data points distant from one another represent the materials with clearly different odor-sensing characteristics. A more numerical approach is to calculate a discrimination factor defined as a linear combination of contribution of each data point to Factors #1 and #2.

Results of such analysis allowed us to rank the materials based in their contribution to discrimination among the 6 odorant samples. The ranking is shown in Table 3.

In order to confirm that the selected 21 GC materials have their odor-sensing properties diverse enough to discriminate among the used odorant samples, the non-transposed data matrix was again fed to the PCA routine—firstly the full data set and secondly only the data for the top 8 GC materials in Table 2.

**Figure 4:**
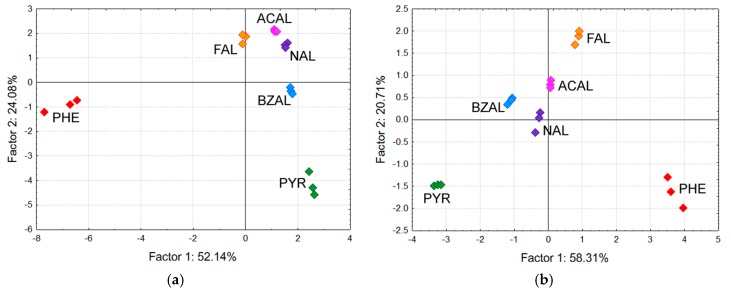
PCA scattering for 6 odorants based on responses of (**a**) all 21 QCM sensors; and (**b**) the 8 top-ranked QCM sensors.

Obtained PCA scattering diagrams are shown in Figure 4a,b, respectively. The labels in the diagrams refer to odorant samples and denote: formaldehyde (FAL), acetaldehyde (ACAL), nonanal (NAL), benzenal (BZAL), 2-phenethylamine (PHE), and pyrrole (PYR). As can be seen in Figure 4a, there is a clear clustering of aldehydes with PHE and PYR data points scattered away from the aldehydes cluster. Also, it seems that the aliphatic aldehydes (FAL, ACAL, NAL) are clustered closer together, leaving the aromatic BZAL slightly at the side. Such result suggests that the 21 GC materials preselected here could be used to discriminate the odorant samples on the basis of (i) functional group; and (ii) aromaticity. In this light, the PCA scattering obtained upon the data set of the 8 top-ranked GC materials should not be significantly worse than the one shown in Figure 4a, i.e., it should at the least show the similar tendencies in discriminating among the odorant samples. Inspection of Figure 4b clearly shows that to be the case—in fact, not only the tendencies seen in Figure 4a remain intact but it seems that clustering/scattering within the aldehydes cluster became slightly better as there clearly is a band along which aliphatic homologues seem to line up. Such a result can be attributed to the apparent redundancy of chemical information carried in the full data set. Nevertheless, the presented results seem to confirm discriminatory power of the GC materials selected in the screening.

### 3.2. Application of GC Materials in Carbon Black Composites

#### 3.2.1. Viability Tests Using 1-channel Chemosensitive Resistor Devices

**Figure 5:**
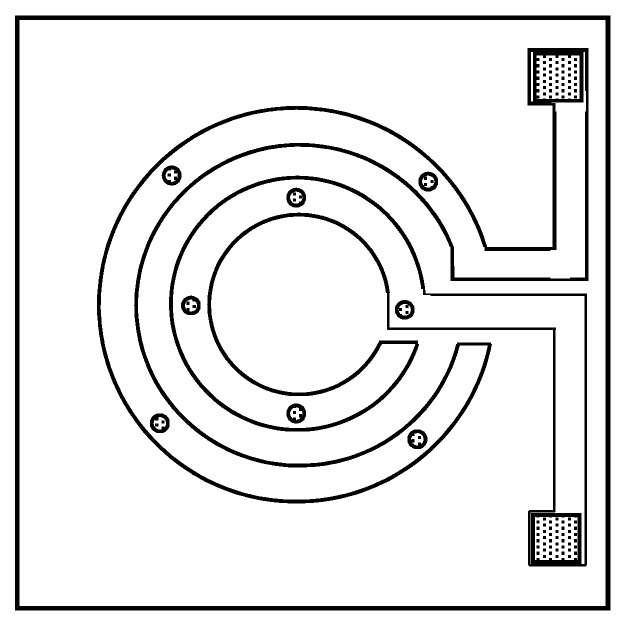
Schematic representation of the 1-channel chemosensitive resistor device used in the initial viability test.

The 21 GC materials ranked in the first part of this study were intended to be used as main interactive part in multi-element chemoresistive devices. In order to evaluate viability of this approach composites of the GC materials with carbon black were first preliminary tested on simple, 1-channel chemoresistive sensors. The sensing device was fabricated on a ca. 4 × 4 mm^2^ chip and is shown schematically in Figure 5.

As mentioned earlier, for this part of the study, the composites were prepared as solutions/suspensions in chloroform. After thorough ultrasonication aiming at the best possible mixing of the particles and GC materials, the composites were coated over the surface of the 1-channel chemoresistive devices. Prior to coating, the devices were pretreated in a plasma cleaner to ensure removal of organic contaminants and appropriate adhesion of the composites to the devices’ surface. The composites were coated onto the electrodes by manual spotting. Although slightly lacking in accuracy and repeatability, this fabrication technique was deemed appropriate for the preliminary evaluation as the purpose here was to test viability of our approach.

**Figure 6:**
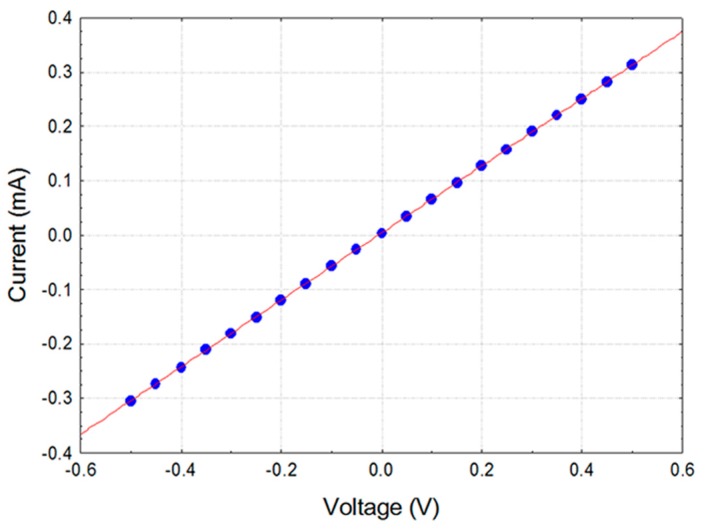
Current–voltage (IV) characteristics for the chemosensitive resistor coated with PEG2000-carbon black composite.

The initial evaluation of the fabricated sensors consisted in checking their IV characteristics. Figure 6 shows results of such evaluation obtained for a device coated with composite containing PEG2000 material. Evidently, there were no deviations from linear character of the relationship. Identical results were obtained for the remaining composites which proved capability of the devices to be used in chemoresistance experiments.

Having proven our chemoresistive devices can be used in vapor sensing experiments, we proceeded with basic evaluation of the fabricated 1-channel chemosensitive resistors in the exposure experiments against vapors of 4 odorants: nonanal (NAL), benzenal (BZAL), 2-phenethylamine (PHE), and pyrrole (PYR). Each sensor was exposed to each sample at 4 different concentrations: 150, 300, 450, and 900 ppb.

**Figure 7:**
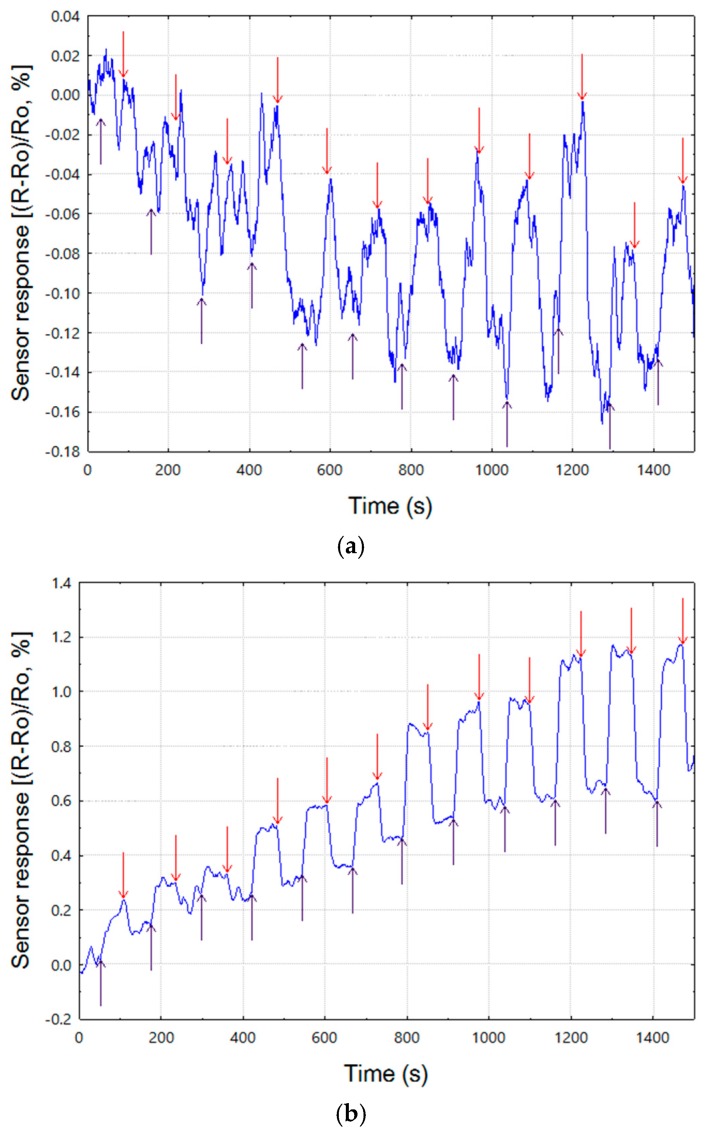
Transient responses of chemosensitive resistors coated with carbon black composites containing (**a**) THEED; and (**b**) PEG20M to vapors of pyrrole (3 triplicates at 4 concentrations).

Example transient responses of sensors with carbon black composites containing THEED and PEG20M GC materials are shown in Figure 7. The results are slightly different from what was expected upon the results of the QCM study, namely, responses of the most sensitive THEED material were not too clear and burdened with considerable noise. Still, evidently the responses at higher concentrations were discernibly clear. On the other hand, the results obtained for PEG20M looked more impressive than expected. The responses were clear even at the lower concentrations though there seem to be an apparent drift of the baseline attributable most likely to temperature variation in the measurement system.

The apparent instability of the THEED composite responses might have stemmed from the relative non-uniformity of the coated composite film, i.e., right after spotting onto the surface we have observed that the carbon black particles seemed to cluster together as the solvent evaporated, making the whole film quite patchy, with regions of high carbon black concentration and ones with virtually no particles present.

In contrast, but consistent with the results shown here, there were no such clustering effects observed for the PEG20M composite. Quite obviously, the problem of uniform distribution of carbon black particles within the GC material matrix had to be addressed in light of our general goal—application of the composites in micro-chips with multi-element chemosensitive resistors.

#### 3.2.2. Viability Tests Using 16-channel Chemosensitive Resistor Devices

The preliminary tests described in the previous section confirmed the potential of using the GC-materials–carbon black composites for odor sensing. In that part of the study, the composites were suspended in chloroform and applied onto the sensing devices manually. Given that the 1-channel devices were relatively large, manual, and not very precise, spotting of the composites was deemed appropriate. However, as the studied composites have been intended to be coated onto multi-element microchips, both uniformity and precision of spotting must have been resolved.

**Figure 8:**
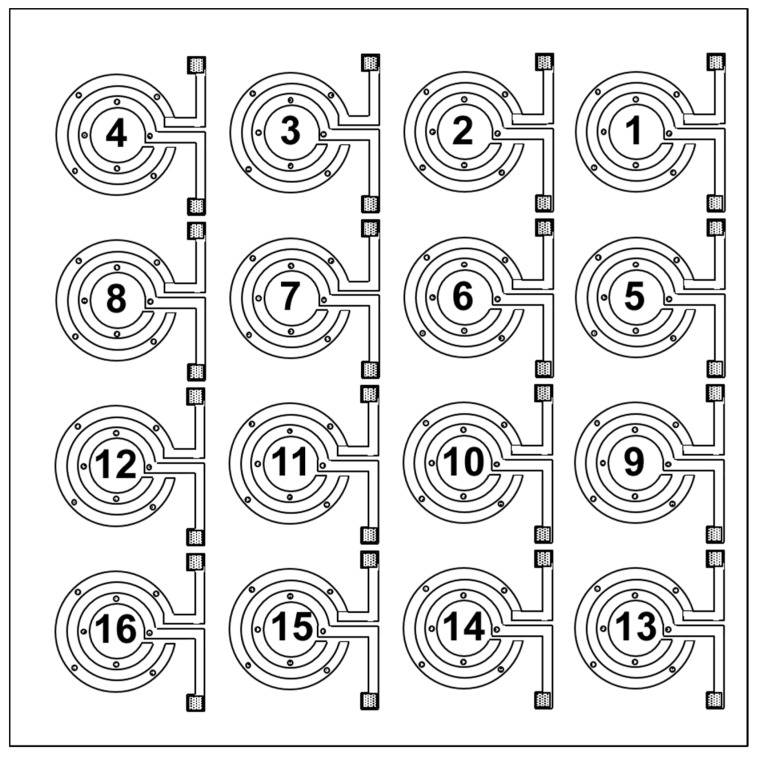
Schematic representation of the 16-channel chemosensitive resistor device used in this part of the study. The numbers denote individual chemosensitive resistor elements coated with appropriate composite (see Table 4 for details).

The chemosensitive microchips used in this part of the study were 16-channel devices shown schematically in Figure 8. The chips were 8 × 8 mm^2^ and the diameter of a spot was ca. 900 µm.

Due to the size of the individual elements as well as a need to more accurately control deposition of the composites we decided to employ for the task a customized spotting machine using micro syringe. As mentioned earlier, one of the problems identified during the preliminary study with 1-channel devices was the fact that a number of composites suspended in chloroform were not developing into a uniform film after spotting, i.e., there were inconsistencies in coverage of the electrodes leading to inconsistent responses when exposed to vapor samples. An effective solution to that problem seemed to be replacing a solvent for the composite materials. We have settled to use DMSO, either pure or mixed with MeCN, as these systems allowed for quite precise spotting and optimum consistency of the resulting composite films in terms of carbon black particle distribution and overall film morphology.

**Figure 9:**
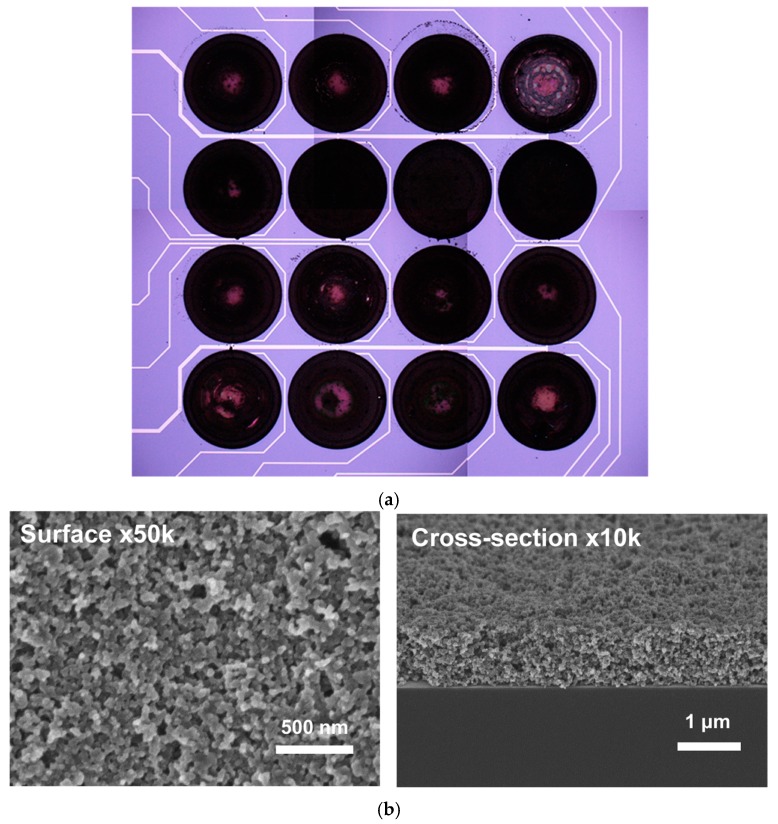
Microscopic images of a 16-channel chemosensitive resistor device: (**a**) optical microscope image of the whole device; (**b**) scanning electron microscopy (SEM) images of surface and cross section of the carbon black—PEG4000 composite film.

In spite of our efforts, a number of materials high in the ranking (Table 3) seemed to cause considerable problems while coated. Those materials were not used for fabrication and were replaced with other available materials in the ranking. A typical microscope image of the fabricated 16-channel device and scanning electron microscopy (SEM) image of the carbon black–PEG4000 composite are shown in Figure 9a,b, respectively.

As can be seen in Figure 9a, there were apparent differences between particle distributions within deposited composite films. The lighter spots in the center of the elements signified areas with lower density of carbon black particles. This, however, didn’t influence the sensing properties of the individual elements as the electrodes were still quite well covered with the composite films which seems to be evident in SEM images shown in Figure 9b.

Characteristics of the final 16 composite suspensions used for coating the microchips are listed in Table 4.

The fabricated microchip sensors were subsequently evaluated in the exposure experiments against vapors of 4 odorants: nonanal (NAL), benzenal (BZAL), 2-phenethylamine (PHE) and pyrrole (PYR). Each sample was presented 3 times at 4 different concentrations: 150, 300, 450, and 900 ppb. The carrier gas was dry nitrogen at room temperature.

**Figure 10:**
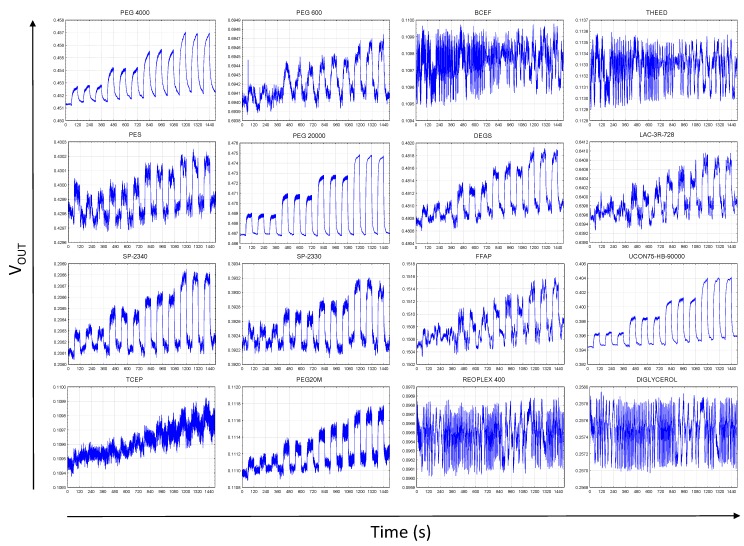
Transient responses of 16-channel chemosensitive resistor microchip sensor to pyrrole (carrier gas: dry nitrogen).

An example of transient responses recorded for PYR is shown in Figure 10. As can be seen, out of 16 sensing elements 12 responded in a clear manner. What has already been observed in the 1-channel experiments, some of the materials expected to respond well based on the QCM study did not seem to respond at all (e.g., THEED). On the other hand, a number of composites seemed to be over-performing as compared to the QCM study. That slight reverse in the expected versus observed results will be further studied to optimize performance of the fabricated multi-element sensors. From the practical standpoint, multiplication of the sensing elements on one chip allowed for capturing of a response pattern for each tested sample.

The responses obtained for each of the 4 samples were used to calculate the individual composites sensitivities. The results are listed in Table 5 along with each element’s base resistance.

Evidently, the fabricated sensors seem to be responding quite well though there seemed to be some surprising developments. Zero sensitivities marked for some composites in Table 5 denote either no response or unstable behavior preventing a clear read-out of the responses.

Inspection of the transient responses indicates there being quite a large room for improvement. One such facet would be the noise level. Suppressing the noise should lead to a more crisp signal and in turn considerable enhancement of the recorded responses. As one possible way to achieve it we consider thermal treatment of the fabricated devices, expected to lead to better organization and stability of the composites.

**Figure 11:**
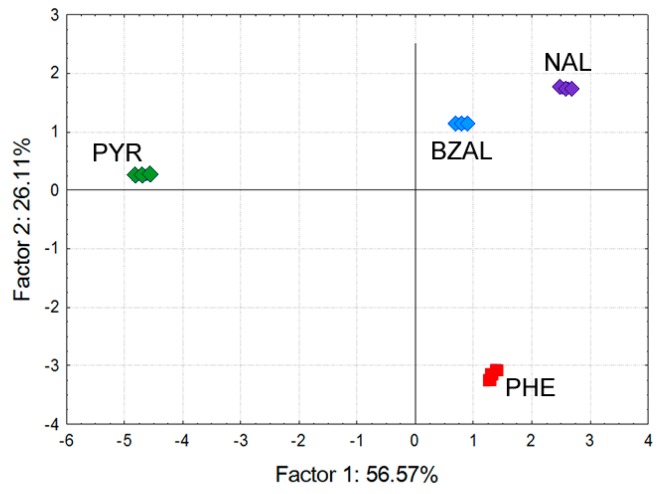
PCA scattering of the 4 odorant samples at 900 ppb obtained upon responses of the 16-channel chemosensitive resistor microchip sensor.

Similar to the QCM study, we used the obtained responses to evaluate the capability of the fabricated sensing devices to discriminate among the vapor samples. The responses at the highest concentration were fed to the PCA routine and the obtained PCA scattering diagram is shown in Figure 11. The labels in the diagram refer to odorant samples and denote: nonanal (NAL), benzenal (BZAL), 2-phenethylamine (PHE), and pyrrole (PYR).

Inspection of the PCA diagram in Figure 11 reveals the samples were scattered into their respective, quite tight clusters. The distances between the clusters suggest that the discrimination followed functionality of the samples as the two aldehydes’ clusters are definitely closer to one another than to the remaining two samples (a triangle PYR-PHE-aldehydes). The result is quite similar to that obtained upon the QCM sensor data which again suggests the captured chemical information was at a considerable level of redundancy allowing cover for either weakly or non-responding sensing elements.

## 4. Conclusions

The paper herein presented a basic study on selection and application of GC stationary phase materials as the sorptive (non-conductive) part in odor-sensing composites used in chemosensitive resistor-based odor sensors.

The chemosensitive resistors are an extremely promising platform for odor-sensing due to their relatively easy fabrication and potential for miniaturize large arrays of sensing devices within a small chip. Still, in order to employ them for odor sensing, the devices need to be activated by odor-interactive films working in a volumetric regime. To date, this has been achieved by using composites of polymers and conductive particle such as carbon black.

The range of possible odor-interactive materials can be broaden enormously should one consider a large group of stationary phase materials used in gas chromatography. The GC materials used in this study were selected upon screening of the chromatographic indices and subsequent evaluation of the actual odor-sensing properties using QCM sensors. We have shown that such approach can allow for selection of an array of sensing materials potentially customized toward a particular sensing task.

The GC materials can evidently be used as a sorptive part in carbon black composites working in a volumetric regime. Their performance though might be slightly different to that observed in pure form, coated onto gravimetric sensors.

Here, we have used the odor-sensitive composites for fabrication of 16-element chemosensitive resistors. Due to considerably high dimensionality of the obtained data we were able to overcome potential problems associated with perceived underperforming of a number of composites. The obtained results suggest that the proposed sensing devices could be effective in discriminating odor/vapor samples at sub-ppm level. Also, in our concept, the carbon black composite materials are to be used on an individual basis, i.e., each chemoresistive element of the micro array is to be coated with a separate blend with distinctive sensing characteristics. Potential further miniaturization of our array would lead to a greater number of chemoresistive elements, each of which should be precisely coated with a distinct blend (each element as an individual chemoresistive sensor). That would be facilitated on the basis of our effort toward optimization and control upon microspotting of the carbon black composites over chemoresistive elements.

In our future work we aim to address a number of problems identified in the present study. In particular: further analyses of the composite films, their morphology and sensing mechanisms; optimization of carbon black distribution in the coated composites; measurement of sensor responses in elevated humidity as well as against larger number of odorant samples (both organic and inorganic).

## Figures and Tables

**Table 1:** Types of gas sensors usually used in electronic nose systems (rough division).

Sensor Type	Sensing Mechanism	Output; Operating Temperature	Sensitivity	Advantages	Disadvantages
Metal Oxide Semiconductor	Reaction of target gases/vapors with oxygen chemisorbed in sensing layer	Electrical resistance change; 250–600 °C	5–500 ppm	Fast response and recovery, low cost, durability/longevity, simplicity	High operating temperature and power consumption, sensitivity to Sulfur poisoning
Metal Oxide Semiconductor Field Effect Transistor	Changes in the drain-source current and the gate voltage upon interaction with target gases/vapors	Electric field change; 75–200 °C	>0.1 ppm	Low cost, small size, good reproducibility	Baseline drift, require controlled environment
Calorimetric	Oxidation of target gases/vapors	Temperature or heat change; 500–550 °C	10–100 ppm	Fast response, stability, low cost	High operating temperature, risk of catalyst poisoning
Optical	Changes of optical properties upon exposure to target gases/vapors	Light modulation, optical changes; Room temperature	Low ppb to ppm	High sensitivity, durability/longevity, low sensitivity to environmental change	Complex circuitry, low portability, suffer from photobleaching
Quartz Crystal Microbalance	Mass change upon sorption of target gases/vapors	Mass change (frequency shift); Room temperature	1 ng	High sensitivity, good precision, diverse range of sensing materials	Complex circuitry, sensitive to humidity an temperature
Surface Acoustic Wave	Mass change upon sorption of target gases/vapors	Mass change (frequency or phase shift); Room temperature	1 pg to 1 ng	High sensitivity, fast response, diverse range of sensing materials, low cost	Complex circuitry, sensitive to humidity and temperature
Carbon Nanofiber Based	Change of electric/electronic properties upon sorption of target gases/vapors	Typically electrical resistance change; Room temperature	<20 ppm	Excellent sorptive capacity, diversity	High cost, difficult to fabricate, low precision
Conducting Polymer	Volume change upon sorption of target gases/vapors	Electrical resistance change; Room temperature	0.1–100 ppm	Fast response, diverse sensing materials, operate at room temperature, high stability, low cost	Sensitive to humidity and temperature, might suffer from baseline drift and saturation
Carbon Particle Based (e.g., carbon black)	Volume change upon sorption of target gases/vapors	Electrical resistance change; Room temperature	ppb to ppm range	Diverse sensing materials, operate at room temperature, easy to miniaturize, low cost	Sensitive to humidity and temperature, might suffer from baseline drift

**Table 2:** Characteristics of the quartz crystal microbalance (QCM) sensors fabricated for the gas chromatography (GC) material selection.

	Material	Abbreviation	Manufacturer	Frequency Shift (kHz)
1.	Free Fatty Acid Phase	FFAP	Sigma-Aldrich	23.6
2.	N,N-Bis(2-cyanoethyl)formamide	BCEF	Tokyo Kasei	17.6
3.	Poly(ethylene succinate)	PDEAS	Sigma-Aldrich	21.6
4.	LAC-3-R-728 (12% DEGS)	LAC-3	GL Sciences Japan	20.5
5.	Silicone OV-210	OV-210	GL Sciences Japan	10.9
6.	Tetrahydrohyethylenediamine	THEED	GL Sciences Japan	21.4
7.	Silicone OV-275	OV-275	GL Sciences Japan	21.1
8.	Reoplex 400	Re-400	GL Sciences Japan	22.8
9.	Diethylene Glycol Succinate	DEGS	Sigma-Aldrich	22.1
10.	Poly[di(ethylene glycol)adipate]	PDEGA	Sigma-Aldrich	20.4
11.	Diglycerol	DI	Tokyo Kasei	12.5
12.	Silicone OV-17	OV-17	Sigma-Aldrich	11.9
13.	Silicone OV-1	OV-1	Sigma-Aldrich	11.0
14.	Apiezon-L	Ap-L	M&I Materials	25.3
15.	SP-2330	SP-3	Sigma-Aldrich	21.1
16.	SP-2340	SP-4	Sigma-Aldrich	18.7
17.	1,2,3-Tris(2-cyanoethoxy)propane	TCEP	Sigma-Aldrich	4.5
18.	UCON 75-H-90000	UCON	Shimadzu	7.8
19.	Poly(ethylene glycol) 20M	PEG20M	Shimadzu	5.8
20.	Poly(ethylene glycol) 20000	PEG20k	Shimadzu	8.9
21.	Poly(ethylene glycol) 2000	PEG2k	Sigma-Aldrich	7.8

**Table 3:** Ranking of the 21 GC materials based on their contribution to discrimination among the 6 odorants.

Rank	Material	Discrimination Factor
1.	Tetrahydrohyethylenediamine	33.66
2.	DiethyleneGlycolSuccinate	21.72
3.	SiliconeOV-210	12.71
4.	Diglycerol	12.60
5.	Poly(ethyleneglycol)20000	12.16
6.	SP-2330	11.31
7.	N,N-Bis(2-cyanoethyl)formamide	9.16
8.	SiliconeOV-1	9.01
9.	Reoplex400	8.56
10.	UCON75-H-90000	6.77
11.	Poly[di(ethyleneglycol)adipate]	5.83
12.	Poly(ethyleneglycol)20M	5.81
13.	1,2,3-Tris(2-cyanoethoxy)propane	2.71
14.	FreeFattyAcidPhase	2.59
15.	SiliconeOV-275	2.44
16.	SiliconeOV-17	2.43
17.	LAC-3-R-728(12%DEGS)	1.91
18.	SP-2340	1.27
19.	Apiezon-L	0.61
20.	Poly(ethyleneglycol)2000	0.58
21.	Poly(ethylenesuccinate)	0.06

**Table 4:** Characteristics of the composite materials used for coating the 16-channel chemosensitive resistor microchips.

Spot Number	GC Materials	Concentration		Solvent
		GC (mg/mL)	CB (mg/mL)	
1	THEED	10	10	DMSO
2	BCEF	10	10	DMSO
3	LAC-3R-728	10	10	DMSO
4	DEGS	10	10	DMSO
5	PES	10	10	DMSO
6	UCON75-HB-90000	10	10	DMSO
7	TCEP	10	10	DMSO
8	SP-2330	10	10	DMSO
9	SP-2340	10	10	DMSO
10	Diglycerol	10	10	DMSO
11	Reoplex400	10	10	DMSO
12	PEG600	10	10	DMSO
13	PEG4000	10	10	DMSO
14	PEG20K	10	10	DMSO:MeCN = 5:1
15	PEG20M	10	10	DMSO:MeCN = 5:1
16	FFAP	10	10	DMSO:MeCN = 5:1

**Table 5:** Summary of the experimental evaluation of the fabricated 16-channel chemosensitive resistor sensors.

	R_0_ (ohm)	Sensitivity (%/100 ppb)			
		PYR	PHE	NAL	BZAL
Tetrahydrohyethylenediamine (THEED)	5420	0	0.006	0.019	0.159
DEGS	8818	0.015	0.006	0.006	0.144
Diglycerol	4407	0	0.001	0.007	0.028
PEG 20000	8247	0.129	0.039	0.019	0.439
SP-2330	5765	0.015	0.027	0.006	0.089
N,N-Bis(2-cyanoethyl)formamide (BCEF)	23,019	0	0.001	0.004	0
Reoplex 400	1602	0	0.006	0	0.004
UCON 75-H-90000	5824	0.167	0.035	0.008	0.375
PEG20M	1492	0.032	0.052	0.005	0.154
1,2,3-Tris(2-cyanoethoxy)propane	1481	0.007	0.005	0	0
Free Fatty Acid Phase (FFAP)	1803	0.035	0.012	0	0.395
LAC-3-R-728 (12% DEGS)	18,914	0.011	0.004	0.001	0.347
SP-2340	2356	0.023	0.016	0.009	0.110
PEG 2000	7654	0.082	0.021	0.006	0.099
Poly(ethylene succinate)	2378	0.011	0.009	0.012	0.043
PEG 600	38,574	0.005	0.001	0.001	0.006
